# Potassium – a scoping review for Nordic Nutrition Recommendations 2023

**DOI:** 10.29219/fnr.v68.10365

**Published:** 2024-02-07

**Authors:** Ulla Toft, Nanna Louise Riis, Antti Jula

**Affiliations:** 1 Center for Clinical Research and Prevention, Copenhagen University Hospital, Frederiksberg, Denmark; 2 Department of Public Health, University of Copenhagen, Copenhagen, Denmark; 3 Department of Clinical Medicine, University of Turku, Turku, Finland

**Keywords:** potassium, minerals, hypokalaemia, hyperkalaemia, hypertension, nutrition recommendations

## Abstract

Potassium (K) is an essential mineral that is necessary for normal cell and membrane function and for maintaining both fluid balance and acid-base balance. Potassium is furthermore very important for normal excitation, for example in nerves and muscle. It is widely available in several food products, with the most important dietary sources being potatoes, fruits, vegetables, cereal and cereal products, milk and dairy products, and meat and meat products. Potassium deficiency and toxicity is rare in healthy people, but dietary potassium is associated with other health outcomes. Results from observational studies have shown that a potassium intake above 3500 mg/day (90 mmol/day) is associated with a reduced risk of stroke. Similarly, intervention studies provide evidence that this level of potassium intake has a beneficial effect on blood pressure, particularly among persons with hypertension and in persons with a high sodium intake (>4 g/day, equivalent to >10 g salt/day).

## Popular scientific summary

- Potassium is an essential mineral, crucial for cell and membrane function, fluid balance, and acid-base balance.
- The main dietary sources of potassium include potatoes, fruits, vegetables, cereals, milk and dairy, and meat.
- No specific biomarker of potassium status has been established, but repeated 24-h urinary excretion is the most valid indicator of intake.
- Potassium deficiency, hypokalaemia, is rare in healthy individuals.
- Increased intake of potassium lowers blood pressure in particular in people with hypertension or high salt intake, and is associated with a lower risk of stroke.

Potassium (K) contributes to the physiology of human health (1, 2), as it has a critical function in the resting membrane potential. Potassium is the most abundant intracellular cation, and the plasma concentration is strictly regulated within narrow limits by the potassium homeostasis, which is critical for the transmission action in neuronal, muscular, and cardiac tissues. Furthermore, potassium is central for hormone secretion, regulation of systemic blood pressure (BP), renal concentration ability, fluid and electrolyte balance, gastrointestinal motility, acid-base balance, mineralocorticoid action, and vascular tone (3). Hence, potassium is important as the osmotically active element inside the cells that is needed for maintenance of total body fluid volume, acid and electrolyte balance, and normal cell function (4).

Potassium deficiency because of low dietary intake is rare, but hypokalaemia may occur during prolonged diarrhoea or vomiting, or because of the use of laxatives or diuretics (5). The human body can handle high intakes of potassium by regulating the renal excretion and cellular uptake or release. Under normal conditions most ingested potassium is excreted via urine (2). With normal potassium excretion there is no evidence of adverse effects of high dietary potassium intake (6).

The metabolism of potassium is strongly related to that of sodium because of the Na^+^/K^+^ -ATPase pump that maintains their extracellular and intracellular concentrations by pumping out potassium ions into the cells and sodium ions out of the cells (1, 3). Renal sodium excretion has been found to be closely related to potassium intake, whereas sodium intake normally does not seem to influence potassium excretion. Furthermore, potassium is interrelated with calcium. Potassium depletion increases urinary calcium loss whereas potassium supplementation has been found to decrease calcium excretion. Similarly, there is a balance between magnesium and potassium (5).

The aim of this scoping review is to describe the overall evidence for the role of potassium for health-related outcomes as a basis for setting and updating dietary reference values (DRVs) for the Nordic Nutrition Recommendations 2023 (NNR2023) (Box 1).

Box 1 Background papers for Nordic Nutrition Recommendations 2023

- This paper is one of many scoping reviews commissioned as part of the Nordic Nutrition Recommendations 2023 (NNR2023) project (7).
- The papers are included in the extended NNR2023 report but, for transparency, these scoping reviews are also published in Food & Nutrition Research.
- The scoping reviews have been peer reviewed by independent experts in the research field according to the standard procedures of the journal.
- The scoping reviews have also been subjected to public consultations (see report to be published by the NNR2023 project).
- The NNR2023 committee has served as the editorial board.
- While these papers are a main fundament, the NNR2023 committee has the sole responsibility for setting dietary reference values in the NNR2023 project.

## Methods

The review on potassium follows the protocol developed within the NNR2023 project (7). The sources of evidence used in the scoping review follow the eligibility criteria described previously (7).

The search was conducted on 14 September 2021 in PubMed, and contained the following terms (“potassium”[MeSH Terms] OR dietary potassium[MeSH Terms]) AND (“2011”[Date – Publication] : “3000” [Date – Publication]) AND review[Publication Type] AND Humans[Filter]. Two qualified systematic reviews, conducted by Newberry et al. and Aburto et al., respectively, were identified and included as the main source of evidence in this scoping review (1, 2, 8).

## Physiology

Potassium is a reactive alkali metal. It is found as salt in nature, and 1 mmol of potassium is equivalent to 39 mg (3). The body absorbs around 90% of the ingested potassium through the gut (9). The estimated average potassium content of an adult human is approximately 135 g in a 70 kg adult (6). Most potassium is found inside cells (~98%), and potassium is the main intracellular electrolyte, with an intracellular concentration of ~150 mmol/L (5.9 g/L). The remaining 2% is found in the extracellular compartment. Thus, potassium is an osmotically active element inside the cells whereas sodium and chloride are main elements outside the cell. The concentration of potassium inside cells is approximately 30 times higher than the concentration in the extracellular fluid. This gradient is maintained by the enzyme Na^+^/K^+^ -ATPase that pumps potassium ions into the cells and sodium ions out of the cells. The plasma potassium concentration is closely regulated at a level around 3.5–5 mmol (137–195 mg)/L by the potassium homeostasis that consists of multiple mechanisms (10, 11). The gradient across the cell membrane and the strictly regulated plasma concentration of potassium is important for broad array of important physiological processes including the transmission of electrical activity in nerve fibres and muscle cells, the resting cellular membrane potential, and the transport of substances across membranes (12). Furthermore, in the endothelial and vascular smooth muscle cells, potassium can affect the contractile state which in turn may influence blood flow and BP (13). Potassium also affects BP by increasing the renal sodium excretion (14). Moreover, potassium plays an important role in regulating the osmotic pressure, and in maintaining fluid balance and regulating the acid-base balance (9), gastrointestinal motility, renal concentration, hormone secretion, and is furthermore a cofactor for many enzymes and is required for the pancreatic secretion of insulin (3).

Mechanisms that help maintain potassium homeostasis include shifting potassium between intracellular and extracellular fluid (internal balance) and retaining or excreting potassium, primarily through the kidneys (external balance) (15). The kidneys is the major organ responsible for the external balance, and around 90% of daily potassium intake is excreted in the urine, whereas the remaining 10% is lost from the gastrointestinal tract and sweat (12). Several endogenous and exogenous factors influence the transfer of potassium between extracellular and intracellular compartments. Increase in plasma concentration of potassium, insulin, epinephrine and aldosterone, metabolic alkalosis, and drug activating β-2 adrenergic receptors increases the uptake of potassium in cells. Whereas, decreased plasma potassium concentration, metabolic acidosis, hyperosmolarity of the extracellular fluid, and α-antagonist drugs will promote transport of potassium from the cells (3).

## Assessment of nutrient status and intake

The homeostatic mechanisms that help maintain plasma potassium in a narrow range makes the plasma potassium concentration inappropriate to assess nutrient status, and there is currently no sensitive or specific biomarker to determine potassium status (16).

Dietary intake can be assessed by self-reported measures using food frequency questionnaires (FFQ). A more valid measure however, is repeated 24 h urinary collection (17).

## Dietary intake in Nordic and Baltic countries

Potassium is widely available in several foods. The most important sources in the Nordic and Baltic countries include potatoes, fruits, vegetables, cereal and cereal products, milk and dairy products, and meat and meat products. Food processing reduces the potassium content of food and drinks, and diets rich in processed food and with a low content of fruits and vegetables are therefore often low in potassium (2). The average dietary potassium intake estimated from National dietary surveys among adults is 3.9 g/day in men and 3.2 g/day in women in Denmark (18), 4.0 g/day in men and 3.4 g/day in women in Finland (19), 3.4 g/day in men and 2.6 g/day in women in Iceland (20), 4.2 g/day in men and 3.4 g/day in women in Norway (21), 3.4 g/day in men and 2.9 g/day in women in Sweden (22), 3.8 g/day in men and 3.0 g/day in women in Estonia (23), 3.0 g/day in men and 2.5 g/day in women in Latvia (24, 25), and 2.9 g/day in men and 2.3 g/day in women in Lithuania (26).

## Health outcomes relevant for Nordic and Baltic countries

### Deficiencies

Depletion of potassium from the body and low blood potassium (hypokalaemia) is most often caused by excessive losses in the urine and gastrointestinal tract, but may also result from an inadequate intake, transcellular shifts or use of diuretics (27). Hypokalaemia is defined as a plasma potassium concentration below 3.5 mmol/L, while severe and life-threatening hypokalaemia is defined by levels below 2.5 mmol/L. Symptoms of deficiency include weakness, fatigue, constipation, and arrhythmias, which impairs the hearts ability to pump blood (27). Deficiency because of inadequate potassium intake is uncommon because of the widespread occurrence of potassium in foods. Loss of body potassium was reported to be prevented with an intake around 39.2 mg/kg body weight/day in one study (28), while another study reported that an intake of 1.6 g/day (40 mmol) was required to avoid loss of potassium and low blood potassium (29). Nevertheless, the lack of a sensitive and specific biomarker to estimate potassium status and the sparse evidence from balance studies does not provide sufficient evidence to draw conclusions about the lower intake level.

### Toxicities

High blood potassium (hyperkalaemia) rarely affects healthy people, but can result from excess dietary intake, intracellular shifts, and impaired potassium excretion (12). Hyperkalaemia is defined as a plasma potassium concentration above 5.0 mmol/L (195 mg/L). Mild to moderate hyperkalaemia are often asymptomatic, but more severe hyperkalaemia with levels above 6.5 (254 mg/L) mmol/L may cause muscle weaknesses, paralysis and cardiac arrhythmias (30). Hyperkalaemia following excessive intake is rare, because of the efficient increase in urinary potassium excretion in persons with normal kidney function. Studies have demonstrated that plasma potassium levels are kept within the normal range after dietary potassium intake of approximately 15 g/day (31, 32). Results from a meta-analysis also reported that intake of potassium supplementation between 1 and 5 g/day (22–140 mmol), in addition to dietary intake, had no adverse health effects in apparently healthy adults (33). Although some of the included studies reported gastrointestinal disorders, including abdominal pain, nausea, and vomiting, it was not possible to identify the dose of potassium at which these symptoms developed. Extremely high doses of potassium supplementation have in some case studies been shown to result in potassium intoxication (34). Case studies have reported deaths after extremely high doses of potassium [see reference 34 for further details (34)]. Based on the current evidence, it has not been possible to set an upper intake level (UL).

### Hypertension

The qualified systematic review by Aburto et al. was initiated by World Health Organization (WHO) and included 35 studies in the assessment of the effect of increased potassium intake on BP [22 randomised controlled trials (RCT) and 11 cohort studies in adults, one RCT and one cohort study in children] (2). They concluded that there is high quality evidence that increased potassium intake reduces BP in people with hypertension. On average, increased potassium intake reduced systolic BP (SBP) by 5.93 (95% confidence interval [CI] 1.70–10.15) mmHg and diastolic BP (DBP) by 3.78 (1.43–6.13) mmHg (2).

The more recent qualified systematic review was commissioned by the National Academies of Sciences, Engineering, and Medicine (NASEM) and conducted by The Agency for Healthcare Research and Quality, USA (1). It included 26 trials (15 parallel RCT; 9 crossover RCT; 2 controlled clinical trials). Similar to Aburto et al., they concluded that potassium supplements reduced BP among adults with prehypertension or hypertension. In a meta-analysis, Newberry et al. pooled results from 18 RCTs conducted among adults (1). The achieved differences in potassium intake between the supplemented group and placebo group ranged from 17 to 120 mmol/day. The analysis showed a significant beneficial effect of potassium supplementation on both SBP of –6.43 mmHg (95% CI: –11.06, –1, 80) and diastolic DBP of –3.50 mmHg (95% CI: –6.10, –0.89). The effect was larger among prehypertensive and hypertensive participants (SBP: –6.95, 95% CI: –12.59, –1.30, DBP: –3.55, 95% CI -6.68, -0.42), compared to normotensive participants, where the effect was smaller and non-significant (SBP: –4.42, 95% CI: –13.85, 5.02, DBP: –3.35, 95% CI: –12.79, 6.10). Several other meta-analyses have shown similar results (2, 16, 35–39). Overall, no evidence was found in the two qualified reviews for an effect in normotensive persons (1, 2).

Most RCTs used supplements to increase potassium intake, while only four RCTs have used a dietary intervention (40–43). Two of the dietary intervention studies found no effect on BP following either a diet enriched in fruit and vegetables or a coaching programme about dietary choices in combination with food vouchers to increase potassium intake (40, 41). In contrast, one study providing dietary counselling showed a reduction in BP in proportion to the achieved decrease in the sodium-to-potassium ratio (42). The fourth study found no differences in BP, but participants receiving dietary counselling reduced their use of anti-hypertensive medication (43). By increasing the potassium intake through dietary changes there is most likely a concomitant change in the intake of other nutrients, which makes it difficult to assess the individual effect of a higher potassium intake.

Based on the current evidence, a potassium intake between 3,500 and 4,700 mg/day seems to result in the greatest BP lowering effect, and there does not seem to be additional benefits of an intake above approximately 4,700 mg/day (2, 35, 38). Yet, a clear dose-response relationship between potassium intake and BP has not been shown. This could be because of differences in the duration of studies, initial BP, methods used to estimate potassium intake, age, baseline potassium intake, habitual diet, baseline sodium intake and race/ethnicity. For example, a meta-analysis by Filippini and colleagues demonstrated that there was a greater BP lowering effect when baseline potassium intake was below 90 mmol/day (3,500 mg/day) compared to a baseline intake above 90 mmol/day (35). Moreover, participants with a sodium intake >4 g/day (>10 g salt/day) had a substantial greater impact of potassium supplementation on BP (SBP of -6.13, 95% CI: –8.42, –3.84 and DBP of –5.30, 95% CI: –8.84, –1.76) compared to participants with a sodium intake <2 g/day (<5 g salt/day) (–2.00, 95% CI: –14.10, 10.10 and no effect on DBP) (35). Correspondingly, the meta-analyses by Aburto et al. and Whelton et al. identified an enhanced BP lowering effect of potassium at high intakes of sodium (39, 44). The potential moderating effect of ethnicity have also been examined in two studies (45, 46). Svetkey and colleagues provided hypertensive participants in the intervention group with 120 mmol/day (4,700 mg/day) potassium for 2 months and reported a larger reduction in BP in black participants (SBP: -20.0, 95% CI: –41.7, –1.7; DBP: –13.0, 95% CI: –22.8, -3.2) compared to the overall group (SBP: -6.3, 95% CI: –11.5, –1.1; DBP: –2.5, 95% CI: –5.4, 0.4)(45). However, the results are inconclusive, because the subgroup of black participants was small (five in the intervention and seven in the control group), and the mean baseline BP was markedly higher among the five intervention participants (156/98 mmHg) than the seven control participants (136/96 mmHg). In contrast, the Trials of Hypertension Prevention (TOHP-I) found no differences between black and white normotensive adults in the lack of effect of increased potassium intake on BP (46).

Most of the RCTs using supplements to increase potassium have used potassium chloride, while a few studies have used other types of potassium salts including potassium bicarbonate, citrate, magnesium citrate, gluconate and aspartate. The results are conflicting with respect to any differential effect on BP (47–51), and based on the current evidence it is not possible to evaluate if one type of supplement is superior compared to others.

There is limited evidence on the effect of increased potassium intake on BP in children (1). A meta-analysis including three RCTs was conducted by WHO in 2012 (52). A total of 326 boys and girls with an average age of 13 years was included in the analysis. Potassium intake in the increased potassium group averaged to 95 mmol/day compared to 57 mmol/day in the lower potassium group. The pooled estimate showed that an increased potassium intake resulted in a non-significant decrease in SBP of –0.28 mmHg (95% CI: –1.05, 0.49) and DBP of –0.92 mmHg (95% CI: –2.00, 0.16).

The two qualified systematic reviews distinguished only between adults and children, and the effects in elderly persons was not specified.

### Cardiovascular diseases

The relationship between potassium intake and risk of cardiovascular diseases (CVD) has been investigated in several observational studies (53–80). A meta-analysis conducted in the qualified systematic review by Aburto et al. found no significant effect of a higher potassium intake on CVD, although the estimates pointed towards a protective effect, with risk ratio (RR) of 0.88 (95% CI: 0.70, 1.10) (2). Accordingly, the qualified review by Newberry et al. concluded that there is insufficient evidence to identify associations of potassium intake with long-term CVD and other chronic disease outcomes of interest, primarily because of the limitations in the potassium intake assessments (1). However, the relationship between potassium intake and risk of total CVD has subsequently been examined by Ma and colleagues that recently conducted a meta-analysis of six prospective cohorts including 10,709 participants with a median follow-up of 8.8 years (81). They found that in the highest quartile of potassium excretion, there was a significant lower risk of CVD compared to the lowest quartile of potassium excretion with a hazard ratio (HR) of 0.69 (95% CI: 0.51, 0.91). Moreover, an increase in potassium excretion of 1,000 mg/day was associated with an 18% lower risk of CVD (HR: 0.82, 95% CI: 0.72, 0.94). The meta-analysis by Ma et al. deviated from the earlier reviews by only including studies in which potassium intake was estimated by at least two 24-h urine samples. The earlier meta-analyses included studies in which potassium intake was estimated from either a single or repeated 24-h urine excretions, 24-h dietary recalls or FFQ. Several 24-h urine collections are the golden standard when estimating potassium intake, which makes the validity of the results from the meta-analysis conducted by Ma and colleagues superior to the earlier meta-analyses.

In addition to the observational studies, two RCT studies have been conducted. The first intervention study included a total of 1,981 male veterans from a retirement home in northern Taiwan. The men were block-randomised by the kitchen in which they had their meals to receive potassium-enriched salt substitute (49% sodium chloride, 49% potassium chloride, 2% other) or sodium chloride and followed them for approximately 2 to 3 years. The results showed a significantly lower age-adjusted rate of CVD-related mortality among men who received the potassium-enriched salt substitute (RR 0.42, 95% CI 0.27, 0.66), which translated to an additional 4 to 11 months of life (82).

A more recent cluster RCT investigated the effect of a potassium-enriched salt substitute on CVD (83). A total of 20,995 participants (from 600 villages in China) either with a history of stroke or being ≥60 years with high BP were included in the study. Villages were randomly assigned to use salt substitute (75% sodium chloride and 25% potassium chloride) or regular salt (100% sodium chloride). The mean follow-up period was 4.74 years. The baseline 24-h potassium excretion was extremely low at 36 mmol/L (equivalent to 1,400 mg of potassium). The baseline 24-h sodium excretion was 187 mmol/L (equivalent to 4.3 g of sodium and 10.9 g of salt). Compared to the regular-salt group, the salt-substitute-group had 20.6 mmol higher 24-h potassium excretion (equivalent to 803 mg of potassium) and 15.2 mmol lower 24-h sodium excretion (equivalent to 0.35 g of sodium and 0.9 g of salt) at follow-up, and had a lower rate of stroke and major CVD events (rate ratio: 0.86, 95% CI: 0.77, 0.96 and 0.87, 95% CI: 0.80, 0.94, respectively) (83).

### Total mortality

Both the qualified review by Aburto et al. (2) and Newberry et al. (1) concluded that there is insufficient evidence to draw conclusions regarding the effect of increased potassium intake and total mortality.

The relationship between potassium intake and total mortality has been examined in two intervention studies, described above (82, 83) and several observational studies (54, 60, 65, 71, 74, 75, 79, 84). Both intervention studies examined the effect of a potassium-enriched salt substitute, which was given to the experimental group while the control group received regular salt (83). Both studies demonstrated a protective effect when using the potassium-enriched salt substitute with one study reporting a HR of 0.90 (95% CI: 0.79, 1.06) (82) and the other reporting a rate ratio of 0.88 (95% CI: 0.82, 0.95) (83). Several observational studies have also identified a protective effect of potassium intake on all-cause mortality with reported lower all-cause mortality risk between 10 and 29% (60, 71, 74, 75, 79, 84). However, Aburto et al. (2) and Newberry et al. (1) concluded that there is inconsistent findings and insufficient evidence to draw conclusions regarding the effect of increasing potassium intake on total mortality.

## Requirement and recommended intakes

Potassium is known to have key functions in nerve stimulation, muscle contraction, fluid balance and acid-base balance. For healthy persons both potassium deficiency and toxicity are uncommon. However, dietary potassium is related to other health outcomes, as potassium appears to have a beneficial effect on BP and to be associated with a reduced risk of CVD.

The reference values (DRV) on potassium from NNR2012 was 3,500 mg/day (90 mmol/day) for men and 3,100 mg/day (80 mmol/day) for women. This also included pregnant and lactating women. The recommendations for children and adolescents were extrapolated from the recommendations in adults and on needs for growth and adjusted for body weight. The recommended intake for children was 1,800 mg/day for children aged 2–5 years, 2,000 mg/day for children aged 6–9 years and 2,900 mg/day for girls, and 3,300 mg/day for boys aged 10–13 years.

Contrary to NNR2012, the most recent recommendation from EFSA concludes that it is unnecessary to give sex-specific values, and therefore the DRV for potassium is set to 3,500 mg/day for both men and women, including pregnant women (85). DRV for lactating women is set a little higher (4,000 mg/day). This is because of the loss of potassium secreted in breastmilk (85).

The most recent DRVs from NASEM, on the other hand, recommend sex-specific DRVs. The committee have set DRVs to 2,600 mg/day for women and 3,400 mg/day for men (16). Recommended DRVs for adult pregnant and lactating women is 2,900 mg/day and 2,800 mg/day, respectively.
